# Hepatitis A and E among immigrants and refugees in Central Brazil

**DOI:** 10.11606/s1518-8787.2022056003839

**Published:** 2022-04-13

**Authors:** Grazielle Rosa da Costa e Silva, Thaynara Lorrane Silva Martins, Carla de Almeida Silva, Karlla Antonieta Amorim Caetano, Megmar Aparecida dos Santos Carneiro, Bruno Vinicius Diniz e Silva, Leonora Rezende Pacheco, Livia Melo Villar, Vanessa Salete de Paula, Regina Maria Bringel Martins, Sheila Araújo Teles

**Affiliations:** I Universidade Federal de Goiás Programa de Pós-Graduação em Enfermagem Goiânia GO Brasil Universidade Federal de Goiás. Programa de Pós-Graduação em Enfermagem. Goiânia, GO, Brasil; II Universidade Federal de Goiás Faculdade de Enfermagem Goiânia GO Brasil Universidade Federal de Goiás. Faculdade de Enfermagem. Goiânia, GO, Brasil; III Universidade Federal de Goiás Instituto de Patologia Tropical e Saúde Pública Goiânia GO Brasil Universidade Federal de Goiás. Instituto de Patologia Tropical e Saúde Pública. Goiânia, GO, Brasil; IV Universidade Federal de Goiás Programa de Pós-Graduação em Medicina Tropical e Saúde Pública Goiânia GO Brasil Universidade Federal de Goiás. Programa de Pós-Graduação em Medicina Tropical e Saúde Pública. Goiânia, GO, Brasil; V Fundação Oswaldo Cruz Instituto Oswaldo Cruz Laboratório de Hepatites Virais Rio de Janeiro RJ Brasil Fundação Oswaldo Cruz. Instituto Oswaldo Cruz. Laboratório de Hepatites Virais. Rio de Janeiro, RJ, Brasil

**Keywords:** Refugees, Latin America, Hepatitis A, epidemiology, Hepatitis E, epidemiology, Seroepidemiologic Studies

## Abstract

**OBJECTIVE:**

To estimate the prevalence of hepatitis A virus (HAV) and hepatitis E virus (HEV) among immigrants and refugees in Goiás, Central Brazil.

**METHODS:**

Overall, 355 individuals were interviewed, and blood samples were tested for anti-HAV and anti-HEV IgG. Anti-HEV-positive samples were similarly tested for HEV RNA.

**RESULTS:**

All participants were from Latin American countries, most of whom, young adult males. The overall anti-HAV IgG prevalence was 87.4% (95%CI: 83.5–90.4), of whom 94.9%, 75.6%, and 60% were from Haiti, Venezuela, and other Latin American countries, respectively (p < 0.001). Age above 19 years and more than 36 months residing in Brazil were associated with a higher prevalence of previous HAV and HEV infection, respectively. Of the children eligible for HAV vaccination according to the National Immunization Program, only eight (44%) had been vaccinated. The overall anti-HEV IgG prevalence was 6.5% (95%CI: 4.4–9.5). All anti-HEV IgG-positive individuals were Haitians, including a child born in Brazil. HEV RNA was detected in two of the anti-HEV IgG-positive samples.

**CONCLUSION:**

The survey detected a high prevalence of anti-HAV and anti-HEV IgG among immigrants and refugees, and active HEV infection among some Haitian participants. Prevention measures are urgently required to interrupt enteric virus transmission in this emergent and vulnerable population.

## INTRODUCTION

According to the United Nations, there were 272 million international migrants worldwide in 2019, and approximately 80 million people were forcibly displaced from their homes, of whom 26 million were refugees^1^. There were an estimated 1.1 million international migrants in Brazil in mid-2020, most of whom were from Latin American and Caribbean countries^2^.

Enteric diseases are important causes of morbidity among migrant populations^3,4^. Therefore, hepatitis A virus (HAV) and hepatitis E virus (HEV) infections, which are generally associated with poverty, overcrowding, and poor sanitation^5,6^, are relatively common among immigrants and refugees^7^.

HAV and HEV are hepatotropic RNA viruses. HAV belongs to the family *Picornaviridae*, genus *Hepatovirus*, species *Hepatovirus* A Virus, and HEV belongs to the family *Hepeviridae*, genus *Orthohepevirus*, species Hepatitis E Virus^12^. Both are responsible for acute self-limited hepatitis; however, HEV can cause chronic hepatitis in immunosuppressed individuals^5,6^. Additionally, pregnant women infected with HEV are at high risk of fulminant hepatitis^6^.

Globally, HEV causes approximately 20 million new cases of hepatitis and 70,000 deaths annually^13^, with nearly 1.5 million new cases of hepatitis A annually and 7,000 deaths. These infections pose the greatest burden on low-income countries^5,6^. While no HEV vaccine is recommended by the World Health Organization^14,15^, available Hepatitis A vaccines are recommended for children aged > 1 year living in intermediate- and low-endemic regions^16^.

Currently, Brazil is an intermediate-endemic region for HAV infection^17^, and offers the recommended HAV vaccine free of charge to all children aged under five years^18^. The HEV prevalence has been observed among different population subgroups. A systematic review estimated a 3% anti-HEV immunoglobin G (IgG) prevalence tin the Brazilian general population^19^.

There are currently no data available on hepatitis A and E among international migrants in Brazil, an emergent and socially vulnerable population. Therefore, this study aimed to estimate the prevalence of HAV and HEV among immigrants and refugees in the state of Goiás, Central Brazil.

## METHODS

There are no official data on the distribution of immigrants and refugees in Goiás. Thus, we obtained personal information from non-governmental organizations assisting immigrants and refugees in Goiás, Central Brazil. According to these non-governmental organizations, Goiania (1.536.097 inhabitants), Aparecida de Goiânia (590.146 inhabitants), and Anápolis (391.772 inhabitants)^20^ receive most international immigrants in Goiás. Therefore, between July 2019 and January 2020, a cross-sectional study was performed among international migrants (immigrants and refugees) residing in these three municipalities, which account for the highest gross domestic product per capita and human development indices (HDI) in the state of Goiás^21^.

In this study, an “international immigrant” was defined as any person migrating from one country to another to establish residence^22^. According to the United Nations High Commissioner for Refugees (UNHCR), an international immigrant chooses to migrate spontaneously for qualification, economic interests, environmental disasters, or other reasons, whereas the refugee is forced to relocate to preserve his freedom, or even his life, due to persecution, armed conflicts, and human rights violations^23^.

The minimum sample size was estimated to be 559 international migrants, assuming a prevalence of 3%^24^, 95%CI (p < 0.05), 80% statistical power (β = 20%), ± 2% precision, and a design effect of 2. However, due to the COVID-19 2019 pandemic, data collection was interrupted after 70% of the target sample had been surveyed. International migrants who had been living in Brazil for approximately 10 years were included in this study. Due to the difficulty of drawing blood from young children, those aged under two years were excluded.

The access to the study population was facilitated by non-governmental organizations and religious groups providing social assistance to international migrants. Data collection was performed in private venues such as churches and non-governmental organization facilities. Individuals were interviewed face-to-face by trained interviewers in the language of their choice (Portuguese, Spanish, French, English, or Creole) to collect socioeconomic data via a common script.

Blood samples were collected and tested for HAV and HEV markers. All samples were tested for total anti-HAV (Elecsys^®^ Anti-HAV II, Roche Diagnostics, USA) by electrochemiluminescence and anti-HEV IgG (Recomwell HEV IgG, Mikrogen GmbH, Neuried, Germany) by enzyme-linked immunosorbent (ELISA). Children aged up to five years were equally tested for anti-HAV IgM by ELISA using Bioelisa HAV IgM assay (Biokit, Barcelona, Spain). Anti-HEV positive samples were tested for HEV RNA using quantitative polymerase chain reaction (PCR) with primers and probes for the ORF three regions of HEV, as described elsewhere^25^.

Data were analyzed using Stata statistical software version 13.0 (StataCorp., College Station, TX). The prevalence of antibodies was estimated with 95%CI. The Chi-squared test and Fisher’s exact test were used to compare categorical variables. To identify variables independently associated with anti-HAV seropositivity, variables with p < 0.1 in the univariable analysis were included in a robust Poisson regression model. The significance level used in the tests was 5%.

This study was approved by the Committee on Ethics in Research of the *Universidade Federal de Goiás* (protocol number: 06871019.7.0000.5083). All participants provided written informed consent. Parents or guardians provided consent for minors, but minors did so if they were old enough. Via a partnership between the project and the health department, hepatitis A vaccines were administered to all eligible children participating in this study.

## RESULTS

In total, we recruited 385 international resident migrants, of whom 355 were eligible, and agreed to participate in the interview and blood draw, the latter being the major reason for non-participation. There were no sociodemographic differences between those who participated and those who refused to. Nine children under 10 years refused to undergo blood draw.

Of the 355 participants, 249 (70.1%) were immigrants, 101 (28.5%), refugees, and five, Brazilian children (1.4%) born to Haitian immigrant parents. Of the total sample, 218 (61.4%) were Haitians, 132 (37.2%), Venezuelans. The others (1.4%) were Colombian (n = 1), Cuban (n = 2), Ecuadorian (n = 1) and Dominican (n = 1). Most of the immigrants and refugees were Haitians (71.5%) and Venezuelans (63.4%) (p < 0.001).

Most migrants were male (56.1%) and self-declared black/brown (85.6%). The median age was 30 years (interquartile range [IQR]: 22–39 years), and the median duration of formal education was 11 years (IQR: 7–14 years). The length of time living in Brazil ranged from < 1 to 120 months (median: 18 months). Among the participants aged 18 years and above, 292 (57.6%) were married, and 178, employed; 86.5% of whom earned R$ 1,500 (equivalent to approximately $375 US) or less per month.

In total, 23 Haitian individuals (6.5%; 95%CI: 4.4–9.5) were anti-HEV positive. Of these, one was a child born in Brazil to Haitian parents. Anti-HEV prevalence among Haitian migrants was 10.6% (95%CI: 7.13–15.33). Individuals who had been in Brazil for longer than 36 months were significantly more likely to have anti-HEV IgG (p = 0.046) (Table 1). Two individuals were HEV RNA positive, but their viral loads were low. All anti-HEV-IgG-positive individuals were also anti-HAV IgG positive.

**Table 1:** Analysis of socioeconomic variables associated with hepatitis A and E seroprevalence among immigrants and refugees in Central Brazil.

Variable	Total	Anti-HAV positive	%	p	Total^a^	Anti-HEV positive	%	P
Country of origin								
Haiti	216	205	94.9		218	23	10.6	< 0.001
Venezuela	127	96	75.6	0.001	132	-		
Other	05	03	60.0		05	-		
International migration category								
Immigrant	251	223	88.8		254	21	8.3	
Refugee	97	81	83.5	0.179	101	02	2.0	0.030
Sex								
Male	196	174	88.8		199	14	7.0	
Female	152	130	85.5	0.366	156	09	5.8	0.631
Age group								
≤ 9 years	23	15	65.2		30	01	3.3	
10–19 years	34	17	50.0	< 0.001	34	-	-	0.182
> 19 years	291	272	93.5		291	22	7.6	
Years of formal education^a^								
Up to 9 years	115	93	80.9		115	7	6.1	
10–12 years	85	78	91.8	0.06	85	5	5.9	0.839
> 12 years	113	100	88.5		113	5	4.4	
Length of time in Brazil (month)								
≤ 6	97	78	80.4		99	02	2.0	
7–18	83	71	85.5	0.004	85	06	7.1	0.036
19–36	67	57	85.1		69	03	4.3	
> 36	101	98	97.0		102	12	11.8	

^a^ Only valid data.

Overall, 18 children were eligible for HAV vaccination, either because they were aged under five years when they arrived in Brazil (n = 13), or were born in Brazil (n = 5). However, only seven of the eligible children (44%), five Venezuelans and two Brazilians had Brazilian records of hepatitis A vaccination. We excluded these seven children from the analysis. The overall anti-HAV IgG prevalence was 87.4% (95%CI: 83.5–90.4). Anti-HAV IgG prevalence was 94.9% (95%CI: 91.1–97.1), 75.6% (95%CI: 67.4–82.2), and 60% (95%CI: 23.1–88.2) among Haitians, Venezuelans, and other Latin American individuals, respectively. We included age, education, length of time living in Brazil, and Haitian origin in a multiple regression model, and only age above 19 years remained independently associated with anti-HAV positivity (both p < 0.001) (Table 2).

**Table 2:** Analysis of multiple factors associated with hepatitis A and hepatitis E seroprevalence among migrant population in Central Brazil.

Variable	Anti-HAV positive		Anti-HEV positive	
	AdjPR (95%CI)^a^	p	AdjPR (95%CI)^b^	p
Haitian origin	1.09 (0.81–1.45)	0.574	-	
Refugee	-		0.29 (0.07–1.26)	1.000
Age > 19 years	1.85 (1.15–2.96)	0.011		
Formal Education > 9 years	0.91 (0.71–1.18)	0.500		
Length of time in Brazil (months)^a^				
≤ 6	1.00		1.00	
7–36	1.04 (0.77–1.41)	0.789	2.50 (0.54–11.62)	0.243
> 36	1.07 (0.76–1.52)	0.685	4.67 (1.03–21.02)	0.046

^a^ Adj PR: Adjusted prevalence ratio; CI: confidence interval;

^b^ Adjusted by Haitian origin, age, education and length of time in Brazil (Goodness of fit = 1); adjusted by international migration category and length of time in Brazil (Goodness of fit = 0.9827).

## DISCUSSION

This is the first study on HAV and HEV among international migrants in Brazil. Most participants were Haitian and Venezuelan. This was unsurprising and reflected the regional migratory influx in the last decade in Brazil and the solidary migratory policy in Latin America and the Caribbean^26^.

Following the 2010 earthquake in Haiti, which caused multiple deaths, loss of homes, and poverty expansion in Haiti, Brazil granted a permanent visa to reunite families on humanitarian grounds to Haitians who could prove domicile in Haiti and had no criminal record^27^. Brazil recognized Venezuelans as refugees under the extended refugee definition in the 1984 Cartagena Declaration on Refugees and the national legislation which also facilitated their migration^1^.

A recent systematic review estimated a high prevalence of HAV infection in Haiti, and an intermediate one in Venezuela^28^. This study observed a high HAV endemicity among all migrants. Almost all individuals aged above 19 years had previously shown HAV infections. However, Haitian participants had the highest HAV prevalence. This finding most likely reflects the lower socioeconomic status of the Haitian population, which has experienced economic and political crises in addition to environmental disasters.

Young children tend to develop asymptomatic hepatitis A and play a key role in disseminating HAV in highly endemic regions^5^. Therefore, surveillance of hepatitis A should be a public health priority among migrants from highly endemic regions migrating into low to intermediate-endemicity countries, such as Brazil^17,28^. Eighteen children in the sample were eligible for HAV vaccination, but only seven had been vaccinated, indicating that the national health system failed to identify and vaccinate migrant children at a high risk of HAV infection.

As there is no gold standard for measurement, the prevalence of anti-HEV can be under- or overestimated depending on the sensitivity and specificity of the assay. In this study, using the Mikrogen^®^ assay, which has 98.9% sensitivity and 98.5% specificity^29^, we observed a relatively high prevalence of anti-HEV IgG among Haitians, indicating a high level of susceptibility. No other information is available on HEV prevalence in the general Haitian population, but a recent study found a 10.3% anti-HEV IgG prevalence among pregnant Haitian women^30^, consistent with the prevalence observed among the Haitian migrants in this study. Therefore, our findings may reflect the HEV epidemiology in Haiti.

A systematic review estimated a 3% anti-HEV IgG prevalence in the Brazilian general population^19^. However, the prevalence found among Haitians is within the wide range of anti-HEV prevalence in Brazilian subgroups. A study employing the same assay used in this study found that the anti-HEV IgG prevalence ranged from 2.5% among kidney transplant recipients^31^ in Goiás in the Midwest to 23.7% in people living in a small municipality in São Paulo in the Southeast^32^.

Notably, we detected anti-HEV IgG in a child because HEV infection is uncommon among children in low HEV-endemic regions^6^. Furthermore, two individuals who had lived in Brazil for < 6 months were anti-HEV IgG positive, and we observed a statistically significant association between the length of time in Brazil and anti-HEV IgG positivity. Furthermore, two individuals were RNA-HEV positive with low viral loads, indicating recent infections. This suggests the HEV is circulating in Haitian migrants, indicating a need for urgent preventive health measures to interrupt the chain of transmission in this specific group.

The limitations of this study include the convenience sampling, implying that participants in this study may be an atypical sample of international migrants in Goiás, even though the majority of migrants in Brazil come from Latin America. Despite this limitation, our study provides the first data on HAV and HEV among international migrants in Brazil. The number of children in this study was small; hence, the study findings may provide an inaccurate estimate of HAV and HEV infection in the children of migrants. Moreover, data on HAV vaccination only included children whose vaccination was in Brazilian records. We cannot exclude the possibility that these children may have received HAV vaccination in their country of origin, but this is very unlikely because HAV vaccination was excluded from the Haitian and Venezuelan national immunization programs^33^.

This study showed a high prevalence of HAV and HEV infections. It suggests HAV immunization failed to reach children of international migrants, highlighting the high vulnerability of this emergent population. Nevertheless, further studies are necessary to describe the HEV epidemiology among Haitians and determine HAV vaccination coverage among migrants overall.
